# Considerations and Suggestions for the Reliable Analysis of miRNA in Plasma Using qRT-PCR

**DOI:** 10.3390/genes13020328

**Published:** 2022-02-10

**Authors:** Eunmi Ban, Eun Joo Song

**Affiliations:** Graduate School of Pharmaceutical Sciences, College of Pharmacy, Ewha Womans University, Seoul 03760, Korea; emban@korea.com

**Keywords:** qRT-PCR, plasma, miRNA, amplification efficiency

## Abstract

MicroRNAs (miRNAs) are promising molecules that can regulate gene expression, and their expression level and type have been associated with early diagnosis, targeted therapy, and prognosis of various diseases. Therefore, analysis of miRNA in the plasma or serum is useful for the discovery of biomarkers and the diagnosis of implicated diseases to achieve potentially unprecedented progress in early treatment. Numerous methods to improve sensitivity have recently been proposed and confirmed to be valuable in miRNA detection. Specifically, quantitative reverse-transcription polymerase chain reaction (qRT-PCR) is an effective and common method for sensitive and specific analysis of miRNA from biological fluids, such as plasma or serum. Despite this, the application of qRT-PCR is limited, as it can be affected by various contaminants. Therefore, extraction studies have been frequently conducted to maximize the extracted miRNA amount while simultaneously minimizing contaminants. Moreover, studies have evaluated extraction efficiency and normalization of the extracted sample. However, variability in results among laboratories still exists. In this review, we aimed to summarize the factors influencing the qualification and quantification of miRNAs in the plasma using qRT-PCR. Factors influencing reliable analysis of miRNA using qRT-PCR are described in detail. Additionally, we aimed to describe the importance of evaluating extraction and normalization for reliable miRNA analysis and to explore how miRNA detection accuracy, especially from plasma, can be improved.

## 1. Introduction

Circulating microRNAs (miRNAs) are highly stable extracellular molecules that circulate in the bloodstream [1,2]. These circulating miRNAs are approximately 22 nucleotides in length and play an important role in gene regulation by binding to and repressing the activity of specific target messenger RNAs (mRNAs). Profiles of miRNAs in plasma and serum have been found to be altered in cancer and other disease states [3,4]. Numerous studies have reported that specific miRNA expression profiles are associated with pathological conditions such as cardiovascular disease [5], cancer [6] and other diseases [7], which may provide diagnostic and therapeutic value as biomarkers. In previous studies, elevated plasma expression levels of miRNA-499 [8], miRNA-122 [9] and miRNA-155 [10] are known to be associated with AMI, liver injury, and inflammation, respectively. Meanwhile, the plasma expression levels of miRNA-34 [11] and miRNA-23a [12] are known to decrease in solid tumors and lung cancer, respectively.

**Figure 1:**
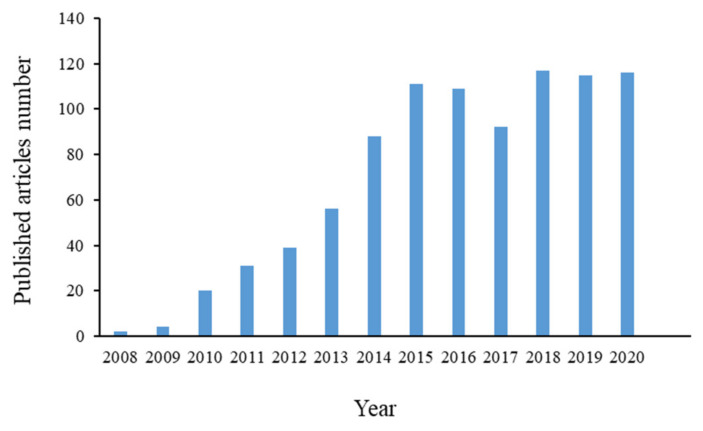
The number of PubMed search results regarding articles reporting on analyses of circulating miRNAs.

Therefore, analyses of circulating miRNAs are important for the discovery and study of disease biomarkers that may aid in disease risk assessment, diagnosis, prognosis, and monitoring of treatment responses (Figure 1).

**Figure 2:**
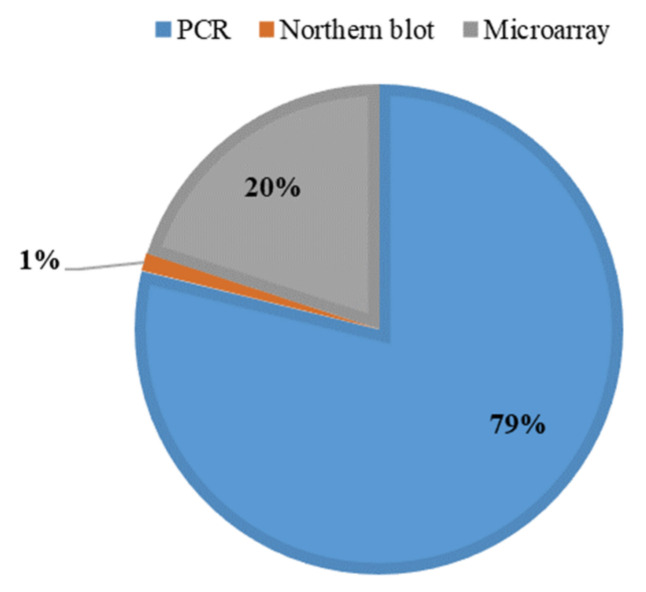
Methods for endogenous miRNA analysis in plasma or serum compared in terms of the proportions of articles reporting their use among PubMed search results from the past 10 years.

Currently, miRNA levels in biological fluids, tissues, and cells are measured after extraction by commercial RNA extraction kits, such as chloroform–phenol-based extraction [13,14], magnetic bead extraction [15], and column-based extraction [16], followed by microarray [17,18], Northern blotting [19,20], and quantitative reverse-transcription polymerase chain reaction (qRT-PCR) analysis [21,22]. Among these methods, qRT-PCR is widely preferred over other detection methods because of its high sensitivity and specificity for detecting low levels of circulating miRNAs in plasma and serum (Figure 2).

However, in previous studies, plasma miRNA levels have varied according to the laboratories performing the measurements [23,24]. This can mainly be due to differences in sample processing, measurements and data analysis [25,26,27,28]. In general, accurate miRNA measurement using qRT-PCR requires a high-quality sample, especially because of the low concentration of miRNA in plasma. Therefore, to increase the extraction efficiency and consistency of miRNAs analysis obtained from plasma and serum, numerous efforts over several years have focused on various highly sensitive and specific methods of miRNA extraction from plasma [29,30,31]. Additionally, similar efforts targeting data normalization [32,33] and the optimization and assessment of PCR conditions have been pursued to improve the accuracy of PCR measurements [34,35]. However, few reviews have focused on the importance and necessity of evaluating PCR conditions for optimizing extraction conditions.

In this review, which focuses on quantity and quality, we describe factors influencing miRNA measurement in plasma and serum using qRT-PCR. Additionally, we review the advantages of assessing PCR efficiency and normalization to obtain reliable and accurate PCR-based results of miRNA analysis in plasma. This evaluation calls attention to the importance of the assessment of PCR efficiency for optimizing PCR and miRNA extraction conditions.

## 2. Inconsistent Measurement of miRNA Extracted in Plasma Using qRT-PCR

Since miRNA analysis using qRT-PCR greatly depends on the quality of the miRNA extract, the results of miRNA analysis are different depending on the sampling procedure applied to the same sample. Many researchers have reported this inconsistency in miRNA analysis results, and various efforts are being made to analyze the factors that cause analysis inconsistency during the sample extraction step [31,36,37,38]. Brunet-Vega et al. compared the Cq levels of miRNAs extracted from the same plasma samples using five commercially available miRNA extraction kits [37]. They reported that the levels of the tested miRNAs were similar, but the levels of the spiked-in exogenous miRNAs were different. For this reason, the analyzed endogenous miRNAs were measured differently from the spike-in exogenous miRNAs. Another research group presented differences in the recovery and levels of miRNAs tested using two different miRNA extraction processes [31]. They also showed that the levels of tested miRNAs were affected by various sample treatments during the sample extraction process. Poel et al. also presented the effect of different carriers and pretreatment times on miRNA extraction recovery and showed inconsistent results between studies [38]. These studies recommended the need for standardization of protocols, including sample handling and extraction processes, to reduce the mismatch results of miRNAs in plasma between laboratories and between assays to perform reliable biomarker screening and discovery of miRNAs in plasma samples.

## 3. Factors Inhibiting Accurate miRNA Measurement in Plasma Using qRT-PCR

miRNA levels in plasma are low—10- and 100-times less than the concentrations in cells and tissues, respectively [39]. Therefore, the reliable and accurate analysis of miRNAs in plasma is a major issue, despite significant developments in the field. Among various analytical methods, qRT-PCR is usually used to analyze circulating miRNA levels in biological fluids, including plasma and serum, owing to its high specificity. However, qRT-PCR analysis can be compromised by various materials, such as matrix and extraction residual reagents in samples, and consequently, miRNA analysis results can vary depending on the purity of the extracted sample. Specifically, the effect of the interference on miRNA analysis in plasma is larger in cells and tissues because of the low abundance of miRNAs in plasma. Therefore, many studies have investigated the interference of miRNA analysis in plasma using qRT-PCR, which is primarily caused by sample components and the residual reagents extracted [36,40].

### 3.1. Sample Matrix

It is established that interference of qRT-PCR analysis is caused by various components present within the sample matrix. Therefore, for reliable qRT-PCR analysis, a high-purity sample from which components, such as proteins, lipids, and carbohydrates have been removed, must be prepared. The abundance of these confounding components varies significantly according to dietary status, anticoagulant type, as well as sampling and storage conditions [41,42]. Besides matrix components, hemolysis can be a major cause of variation in miRNA levels. Several miRNAs are found in large amounts in red blood cells (RBCs), and they are released from RBCs as a result of hemolysis, thereby increasing the level of certain miRNAs in the blood. However, hemolysis is more difficult to control than other conditions because it occurs frequently during blood sampling. Many authors have reported that miRNA qRT-PCR analysis results differ according to the degree of hemolysis of the sample. Myklebust et al. showed that qRT-PCR miRNA measurement is influenced by hemolysis [43]; in their study, the plasma miRNA concentration increased as the hemolyzed proportion of the sample increased, but the degree of increase depended on the miRNA type. Specifically, miR-16 is one of the most abundant miRNAs in RBCs [44], and many studies have shown that hemolysis may increase miR-16 levels in plasma. This is especially important because, due to its high abundance relative to other miRNAs, miRNA-16 is used as an endogenous reference gene to normalize the data after qRT-PCR analysis. Thus, hemolysis must be taken into consideration for accurate screening of blood miRNA levels. Feng et al. also demonstrated the effect of the sample matrix on miRNA analysis using qRT-PCR [36]. They showed variability in miRNA levels among matrices with varying compositions, including in terms of types or levels of anticoagulant molecules, plasma protein, and lipids, and hemolysis was analyzed using qRT-PCR. The authors showed that the factors influencing the sample matrix (leading to variability in miRNA analysis in plasma using qRT-PCR) are primarily associated with dietary status, anticoagulant selection, and plasma sample storage conditions. Mompeón et al. also demonstrated the effect of hemolysis on miRNA analysis using qRT-PCR, observing that miRNA levels differed between serum and plasma [45]. In addition to differences in sample components and conditions, there may be differences in the degree of interference with qRT-PCR analysis due to extraction efficiency. Variations in analysis results may also be associated with the purity of the prepared sample, which depends on the sample extraction method. For this reason, various sample extraction kits and methods have been developed, and some of these have been reported to reduce intralaboratory and interlaboratory variability. Several research groups have presented and compared commercial RNA extraction kits, and studies related to the standardization of miRNA extraction methods from biological fluids have been conducted [46,47,48]. Column-based extraction kits often obtain high-quality miRNA extracts, and they are associated with lower extraction variation chloroform–phenol-based kits. However, chloroform–phenol-based kits, such as Trizol, are also known for favorable extraction recovery and costs.

### 3.2. Residual Reagents

For reliable qRT-PCR analysis, it is necessary to minimize any interfering factors using an extraction approach. For this reason, different extraction methods have been developed and applied to measure miRNA in cells, plasma, serum, and tissues. Currently, commercial miRNA extraction kits are largely divided into chloroform–phenol-based reagent kits and column-based extraction kits. Both methods include phenol extraction, which is a long-established approach to extracting nucleotides from biological samples. However, with phenol extraction, residual solvents, including phenol, remain in the final purified RNA sample after extraction and interfere with qRT-PCR-based miRNA analysis as a contaminant [49]. Among this interference caused by extraction reagents, residual phenol not only interferes with PCR analysis, but it can also cause errors in the quantification of RNA extracted from plasma. Specifically, the interference by residual phenol on miRNA analysis in plasma or serum is severe (relative to interference in cells and tissues) because of the low abundance of miRNAs in plasma and serum. For this reason, many researchers have investigated the issue of residual phenol. Spectrometric overestimations caused by residual phenol from extracted RNA yields have frequently led to inaccurate and variable plasma miRNA measurements. This problem is exacerbated by the fact that the wavelengths of RNA and phenol are similar, and the level of RNA in plasma is low compared with residual phenol. Several companies have, therefore, developed nanodrop systems to measure residual phenol concentrations in extracted samples to prevent mismeasurement of the amount or concentration of extracted RNAs by spectrometry [50,51]. Additionally, researchers have developed new systems that are not based on absorbance but that use specific fluorescent dyes for small RNAs to reliably measure extracted miRNAs [52,53]. For circulating biomarker detection analysis, accuracy could be optimized via the use of equal volume inputs rather than the same amount of RNA [54]. In plasma miRNA analysis by qRT-PCR, instead of direct RNA measurements, extraction recovery and analyzed samples are evaluated and normalized using spiked exogenous miRNA.

## 4. Important Considerations for Reliable miRNA Analysis Using qRT-PCR

Plasma miRNA analysis is an important area of biological and clinical research that is gaining increasing recognition. However, low plasma miRNA levels are associated with miRNA measurement errors and consequent inaccurate analyses [32]. These errors are mainly caused by interference in the sample matrix. Therefore, researchers have attempted to evaluate interference and develop normalization methods to minimize errors caused by such interference.

### 4.1. Amount of miRNA

Given that highly purified samples are needed for successful qRT-PCR analysis, many researchers have concentrated on developing methods that emphasize extracting high-quality RNA rather than high yields. Column-based extraction kits are often used for RNA extraction from various sample types. However, high quantities of RNA are also needed for reliable miRNA analysis, but it is challenging to efficiently extract RNAs from serum and plasma [55]. For this reason, chloroform–phenol-based extraction is still used to extract miRNAs from plasma, although several researchers have discussed the problems associated with chloroform–phenol-based extraction methods in PCR analysis. Various extraction kits have been compared and investigated [53]. In addition to extraction kits, various trials have been conducted, including comparisons between different modifications of extraction processes (such as the addition of carriers to trigger precipitation or the modification of incubation conditions) to increase extraction recovery without loss of quality. Some research teams have optimized carrier types, concentrations, and incubation conditions to maximize plasma miRNA extraction [56,57].

### 4.2. Normalization

**Figure 3:**
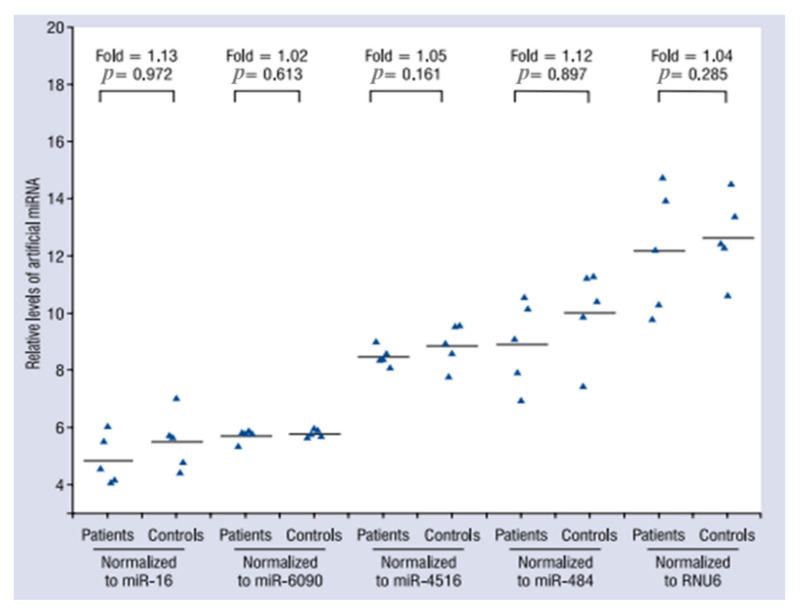
Effects of normalization by different reference gene methods on the expression levels of miRNAs from plasma samples of stable coronary artery disease patients and healthy controls (*n* = 5 in each group). For each group, 2 µL of exogenous cel-miR-39 was spiked into 300 µL plasma. The levels of cel-miR-39 were assessed by qRT-PCR and were normalized to miR-16, miR-6090, miR-4516, miR-484, and RNU6 [62].

Similar to other analytical methods, qRT-PCR analysis is subject to variations or errors, including in association with elements such as sample handling and volume measurements. Specifically, qRT-PCR plasma miRNA analysis can be greatly influenced by nutritional status, anticoagulant type, and plasma storage conditions. Therefore, normalization is important to mitigate variations in qRT-PCR analysis. One of methods for normalization of miRNA analysis by qRT-PCR is global normalization, which uses the calculated mean of all miRNAs in a given sample as the normalizer. This method is highly recommended when dealing with large scale miRNA expression profiling studies where several hundreds of miRNAs are analyzed [58]. However, global normalization cannot be applied for small-scale studies. Another popular method is normalization through reference genes. U6 is a small nuclear RNA commonly used as an endogenous internal control to normalize miRNA expression levels in different biological samples, including plasma. However, there is evidence that U6 plasma levels vary under certain conditions [59]. Therefore, various studies have been conducted to identify more reliable reference genes for normalizing endogenous plasma miRNA levels [60,61]. However, evaluations of different reported reference genes have yielded inconsistent findings [33,62,63,64]. Consequently, efforts have led to the identification of an appropriate endogenous miRNA for normalization, accounting for differences in plasma according to various factors, including disease status, sex, and age. External references, such as cel-miR-39-1 for normalization, are also frequently used to correct for extraction recovery and measurement. Zhang et al. showed the accuracy of normalization by reference gene candidates using exogenous miRNA (spiked-in cel-miR-39) as a target (Figure 3).

Such an approach can eliminate multiple deviations of the experimental process, yielding more robust results. However, the approach also makes the experimental procedure cumbersome, and clinical applications more inconvenient. For example, cel-miR-39 was spiked into serum immediately before RNA extraction, allowing for the control of technical variation. However, the cel-miR-39 recovery was variable, ranging from 1% to 56%, thereby demonstrating the inherent need to take technical variability into account when performing absolute quantification [65]. Additionally, extraction kit-dependent differences in isolation yields across exogenous cel-miRs were reported. Nevertheless, the use of an exogenous cel-miR for normalization and correction presents less variability than strategies based on the concentration of endogenous components, such as the frequently used miR-16-5p [66]. Therefore, the normalization or correction strategy and, to a lesser extent, postanalytical concerns strongly limit the clinical implementation of miRNAs. To date, researchers are yet to establish a robust method of miRNA quantification for qRT-PCR that is clinically easy to implement and universally accepted.

### 4.3. Amplification Efficiency

In each cycle, qRT-PCR automatically detects the PCR amplification of a specific gene target. In PCR analysis, the number of target sequence molecules should double during each replication cycle, corresponding to 100% amplification efficiency. However, in practice, inappropriate reaction conditions and polymerase inhibition affect primer template annealing, resulting in decreased amplification efficiency and potentially leading to inaccurate conclusions. The assessment of factors affecting amplification efficiency provides information regarding inappropriate or suboptimal reaction conditions, as well as the presence of contaminants interfering with accurate qRT-PCR analysis. Therefore, qRT-PCR assays result in significant uncertainty due to variations in amplification and extraction efficiency [67,68]. For these reasons, several studies have investigated amplification efficiency to improve the accuracy and reliability of qRT-PCR analysis [23,37,69,70,71]. Brunet-Vega et al. demonstrated the necessity of exogenous genes through circulating miRNA profiling analysis using a commercial RNA extraction kit and exogenous genes to control for technical factors affecting final miRNA levels. Additionally, the observation that PCR efficiency reduces the variability of miRNAs circulating between samples should be validated because miRNA analysis in plasma using PCR can be affected by samples and PCR components [37]. Lebuhn et al. also demonstrated interlaboratory variation in qRT-PCR miRNA analysis in terms of amplification efficiency according to qRT-PCR-related factors, including interlaboratory differences in extraction steps [23]. Zununi Vahed and our team have successfully optimized extraction conditions through evaluations of amplification efficiency for reliable endogenous miRNA analysis using qRT-PCR [69,70]. Table 1 shows extraction method-dependent differences in amplification efficiency and quantification cycle (Ct) values of extracted miRNA.

Svec et al. reported on factors associated with effective amplification efficiency [71]. Given that polymerase inhibition is caused by contaminants transferred from the RNA isolation process or sample matrix, for factors related to PCR reaction conditions, extraction conditions should be evaluated and optimized through assessments of amplification efficiency to reduce contaminants interfering with accurate qRT-PCR analysis. These studies have revealed that contaminants have a greater effect on qRT-PCR-based miRNA analysis from plasma than samples such as cells and tissues due to low levels of plasma miRNAs. Therefore, potential sources of interference in extracted plasma samples must be identified and corrected based on amplification efficiency before conducting qRT-PCR analysis. Reaction conditions such as annealing and primer conditions must first be evaluated and optimized in terms of amplification efficiency to ensure accurate analysis. Importantly, based on the evaluation of amplification efficiency, the specificity and sensitivity of qRT-PCR results can differ by primer type and concentration [72].

## 5. Discussion

Clinical and pharmaceutical research about plasma or serum miRNAs is becoming increasingly important. Consequently, endogenous plasma miRNA analysis has also become critical. Analysis of endogenous plasma miRNA is conducted using qRT-PCR, but such analyses have shown high variability between different laboratories and individuals [24]. One explanation for this is that miRNA levels in plasma are low, and qRT-PCR analysis is consequently affected by extraction and sample components. Therefore, several studies have investigated reproducible techniques and adjustments applied to miRNA analysis, such as sample extraction and normalization techniques. The high sensitivity of qRT-PCR as an analytical tool is matched by its sensitivity to interference by various factors. Therefore, the optimization of an effective extraction method is a major consideration for reliable PCR analysis, and many published articles report on extraction methods to minimize sample interference. Similarly, normalization and optimization of PCR conditions in terms of amplification efficiency have also been investigated, with consideration of issues, such as hemolysis, as major causes of interference. Specifically, normalization is heavily emphasized as an important factor facilitating reliable evaluation of plasma miRNA. Normally, reference genes are used to normalize endogenous miRNA, while exogenous miRNAs, such as cel-39-1, can also be added to samples to compensate for differences in extraction efficiency between samples [37]. However, these do not reflect extraction recovery because extraction efficiency differs between endogenous and exogenous miRNA, as does the effect of amplification efficiency on the environment of the extracted sample. Therefore, in addition to exogenous miRNA, appropriate reference genes are needed to normalize extracted endogenous miRNA levels, and reference gene selection must be prioritized because some reference genes can differ depending on the sample condition and type. With normalization, the evaluation of amplification efficiency is also crucial for the reliability of qRT-PCR studies [37,73,74]. Sreedharan et al. demonstrated improvements in the reliability of expression data through primer-dependent improvements in amplification efficiency [73]. Variations in primer concentration and annealing temperature, as well as primer design, can affect amplification efficiency and consequently affect the reliability of expression data. Optimization of PCR and extraction conditions through assessments of amplification efficiency might be important determinants of accurate and reliable qRT-PCR analysis of endogenous plasma miRNA.

**Figure 4:**
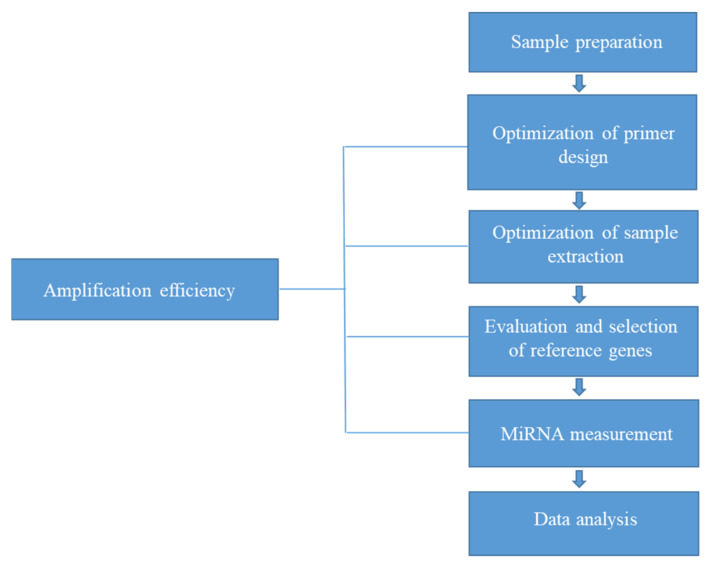
Suggested flowchart for qRT-PCR analysis of plasma miRNA.

This review describes the considerable variation and poor reproducibility of qRT-PCR-based plasma miRNA analysis associated with incomplete optimization of extraction and RT-PCR conditions through amplification efficiency and normalization. In the context of evaluating amplification, the use of exogenous and endogenous reference genes for normalization is necessary for the reliable and reproducible quantification of circulating miRNAs in plasma. These factors should be considered when translating the analysis of circulating miRNAs from plasma and serum into validated biomarker-based tests for routine clinical use. However, despite advances, such as the standardization of extraction processes and normalization for reliable qRT-PCR analysis of plasma miRNA, issues remain regarding the accuracy of qRT-PCR analysis due to individual differences in matrix composition. Therefore, as shown in Figure 4, we propose that the optimization of extraction conditions and the evaluation and identification of dependable reference genes (based on assessments of amplification efficiency) are necessary to ensure reliable and robust qRT-PCR-based miRNA analysis necessity for future applications of circulating miRNAs.

## 6. Conclusions

This review presented factors influencing measurements of miRNAs in plasma/serum including assessment of PCR efficiency and normalization to obtain reliable and accurate PCR-based results of miRNA analysis in plasma. It could suggest the necessity of the assessment of PCR efficiency for the optimization of PCR and miRNA extraction conditions. In this review, the effect of factors related to extraction efficiency such as sample matrix, residual solvent after extraction process and RNA amount was described among various factors influencing measurements of miRNAs using qRT-PCR. These factors may cause inhibitors of qRT-PCR analysis and consequently can lead to inaccurate qRT-PCR analysis. The necessity of amplification efficiency and normalization as another considerable part was reported for reliable and reproducible quantification of circulating miRNAs in plasma using qRT-PCR. From this review, we conclude that the optimization of extraction conditions and selection of reliable reference genes based on assessment of the amplification efficiency should be prioritized for achieving a reliable qRT-PCR-based miRNA analysis in plasma/serum.

## Figures and Tables

**Table 1:** Mean quantification cycle (Ct), PCR efficiency, and correlation–coefficient (*R^2^*) values of miR-21 isolation from cell lines, urine, and plasma by different methods.

Method	Body Fluids				Cell Lines				Urine Sediments			
	Ct	E (%)	*R* ^2^	Slope	Ct	E (%)	*R^2^*	Slope	Ct	E (%)	*R* ^2^	Slope
KCH_3_COOH	31.1 ± 0.4	103.54	0.995	−3.24	17.5 ± 0.07	99.5	0.992	−3.33	23.0 ± 0.3	100	0.998	−3.32
PEG4000	33.2 ± 1.0	111.5	0.993	−3.074	20.0 ± 0.13	95.49	0.996	−3.44	25.7 ± 0.45	98	1	−3.37
PEG6000	36.8 ± 0.2	91.99	0.977	−3.53	18.3 ± 0.32	116	0.983	−2.99	28.1 ± 0.74	86	0.976	−3.683
LiCl8M	34.8 ± 0.5	94.17	0.982	−3.47	21.8 ± 0.49	100.46	0.97	−3.31	31.7 ± 0.02	108	0.961	−3.145
Ethanol+LiCl	33.3 ± 0.07	99.46	0.994	−3.34	20.9 ± 0.9	105	0.998	−3.189	25.3 ± 0.62	114	0.993	−3.024
Ethanol	35.0 ± 0.09	120.02	0.979	−2.92	22.4 ± 0.03	98.03	0.991	−3.37	37.8 ± 0.63	105	0.982	−3.189

Data from three biological replicates of cell lines (HT-29 and HUVEC), body fluids (plasma), and urine samples. (Reproduced from [69]).

## Data Availability

Not applicable.
